# The Digestibility of *Hibiscus sabdariffa* L. Polyphenols Using an In Vitro Human Digestion Model and Evaluation of Their Antimicrobial Activity

**DOI:** 10.3390/nu13072360

**Published:** 2021-07-10

**Authors:** Yassine Oulad El Majdoub, Giovanna Ginestra, Giuseppina Mandalari, Paola Dugo, Luigi Mondello, Francesco Cacciola

**Affiliations:** 1 Department of Chemical, Biological, Pharmaceutical and Environmental Sciences, University of Messina, 98168 Messina, Italy; youladelmajdoub@unime.it (Y.O.E.M.); gginestra@unime.it (G.G.); pdugo@unime.it (P.D.); lmondello@unime.it (L.M.); 2 Chromaleont s.r.l., c/o Department of Chemical, Biological, Pharmaceutical and Environmental Sciences, University of Messina, 98168 Messina, Italy; 3 Department of Sciences and Technologies for Human and Environment, University Campus Bio-Medico of Rome, 00128 Rome, Italy; 4 BeSeps.r.l., c/o Department of Chemical, Biological, Pharmaceutical and Environmental Sciences, University of Messina, 98168 Messina, Italy; 5 Department of Biomedical, Dental, Morphological and Functional Imaging Sciences, University of Messina, 98125 Messina, Italy; cacciolaf@unime.it

**Keywords:** simulated human digestion, digestibility, *Hibiscus sabdariffa*, anthocyanin, polyphenol, antibacterial activity

## Abstract

*Hibiscus sabdariffa* L. (*H.s.*) is a polyphenolic-rich plant commonly consumed either as a beverage or spice. The aim of the present study was to evaluate the in vitro digestibility of *H.s.* polyphenols using an in vitro model of digestion which simulates the human stomach and small intestine. The bioaccessible polyphenols released in the digested samples were analyzed by liquid chromatography coupled to photodiode array and mass spectrometry detection. *H.s.* anthocyanins (cyanidin-3-*O*-sambubioside and delphinidin-3-*O*-sambubioside) content drastically dropped during the digestion process from 2.91 ± 0.03 µg g^−1^ and 8.53 ± 0.08 µg g^−1^ (*w/w*) CG (Cyanidin-glucoside) in the raw extract, respectively, to 0.12 ± 0.01 µg g^−1^ 0.12 ± 0.01 µg g^−1^ (*w/w*) CG at the end of duodenal digestion. Total polyphenols also have shown a decrease from 1192.65 ± 30.37 µg g^−1^ (*w/w*) in the raw extract to 282.24 ± 7.21 µg g^−1^ (*w/w*) by the end of gastric digestion, in contrast to their increase by the end of duodenal digestion 372.91 ± 3.97 µg g^−1^ (*w/w*). On the other hand, the decrease in certain compounds (e.g., caffeoylquinicandcoumaroylquinic acids) was observed during gastric digestion resulting in an increase of quinic acid in the duodenal aliquots, thus suggesting that this compound was derived from the degradation of the more complex hydroxycinnamic acids. *H.s.* extract also exhibited a bacteriostatic effect against *Staphylococcus aureus* ATCC 6538 (MIC of 2.5 mg mL^−1^) and a bactericidal effect against a food isolate of *Listeria monocytogenes* (MBC of 2.5 mg mL^−1^). The undigested polyphenols of *H.s.* in the upper gastrointestinal tract enters the colon, where they are metabolized by the gut microbiota. The present study results showed that resistance of *H.s.* polyphenols during gastrointestinal digestion might affect their uptake, resulting in a decrease in their digestibility.

## 1. Introduction

*Hibiscus sabdariffa* (*H.s.*), which belongs to the family of *Malvaceae,* is commonly known as asroselle, bissap, or karkade. It is widely cultivated in tropical and sub-tropical regions such as India, Mexico, Egypt, and Thailand. Traditionally *H.s.* has been used as a hot herbal or cold drink beverage given its richness in anthocyanins, which are water-soluble flavonoids usually present in their glycosylated forms. [1,2,3,4,5]. Anthocyanins are relevant antioxidants with the effect of boosting many biological functions after ingestion. The two predominantly known anthocyanins in *H.s.* are represented by cyanidin-3-*O*-sambubioside (C3S) and delphinidin-3-*O*-sambubioside (D3S) [6].

In addition to anthocyanins, *H.s.* does contain other numerous bioactive compounds that exert many physiological and pharmacological activities. The in vivo biological activity of all bioactive compounds is firmly conditioned by their availability in their site of action. One of the major limiting factors affecting the beneficial effects of polyphenols is their bioaccessibility and subsequent absorption in the gastrointestinal tract (GIT), together with their bio-transformation by the gut microbiota enzymes [7]. The term ‘bioaccessibility’ is defined as the proportion of a nutrient or phytochemical compound ‘released’ from a complex food matrix during digestion and, therefore, potentially available for absorption in the upper GIT. In previous studies we evaluated the release of lipids, proteins, and polyphenols during simulated human digestion from natural and blanched skins [8] and demonstrated how the food matrix affects the bioaccessibility of polyphenols during simulated human digestion [9]. Furthermore, the evaluation of the bioaccessibility of pistachio polyphenols, xanthophylls, and tocopherols during simulated human digestion demonstrated that a high percentage of polyphenols were released in the gastric phase, whereas the presence of a food matrix (muffin) decreased the bioaccessibility of protocatechuic acid and luteolin [10].

Since *H.s.* polyphenols were previously studied for their colonic fermentation in vivo, specifically focusing on anthocyanins [6], the present study reports on the digestibility of *H.s.* polyphenols using an in vitro model of human gastric and duodenal digestion [11]. Moreover, the evaluation of the antimicrobial effect of the polyphenols-rich extract was evaluated. Given the global increase in antibiotic resistance, more and more efforts are concentrated on the identification of novel bioactives against human pathogens to be used alone or in combination with traditional antimicrobial compounds. The dried polyphenol extracts of *H.s.* were exposed to the in vitro simulated human digestion and all the obtained aliquots during the gastric and duodenal digestion next to the raw extracts were analyzed by high performance liquid chromatography coupled to photodiode array and mass spectrometry detection (HPLC-PDA-MS/MS).

## 2. Materials and Methods

### 2.1. Chemicals and Reagents

LC-MS grade acetonitrile (ACN), methanol (MeOH), trifluoroacetic acid (TFA), ethyl acetate, formic acid, and water were obtained from Merck Life Science (Merck KGaA, Darmstadt, Germany). The employed polyphenols standards for the semi-quantification include cyanidin-3-*O*-glucoside, caffeic acid, quercetin, coumarin, and kaempferol (purity ≥95.0%) and they were purchased from Merck Life Science (Merck KGaA, Darmstadt, Germany). Stock solutions of 1000 mg L^−1^ were prepared for each standard by dissolving 10 mg in 10 mL of methanol.

Chemicals and enzymes employed in the in vitro simulated digestion include the following: sodium chloride (NaCl), potassium chloride (KCl), calcium chloride (CaCl_2_), urea, cholesterol, sodium phosphate monobasic (NaH_2_PO_4_), zinc sulphate heptahydrate (ZnSO_4_·7H_2_O), α-amylase from human saliva type XI (A1031-1KU), egg-phosphatidylcholine (PC, 840051P), pepsin from porcine gastric mucosa (P6887), α-chymotrypsin type II from bovine pancreas (C4129), trypsin type IX-S from porcine pancreas (T0303), lipase type VI-S from porcine pancreas (L0382), colipase from porcine pancreas (C3028), α-amylase type VI-B from porcine pancreas, sodium glycodeoxycholate(G9910), and taurocholic acid sodium salt hydrate (T4009). These were all purchased from Merck KGaA (Darmstadt, Germany). The lipase was a gastric lipase analogue of fungal origin (F-AP15) from Amano Enzyme Inc. (Nagoya, Japan).

### 2.2. Sample and Sample Preparation

The dried calyces of *H.s.* were purchased from a local market in Meknes, Morocco. The botanical identification of the plant materials was performed at the Department of Biology, Faculty of Sciences, Moulay Ismail University, Meknes, Morocco.

**Figure 1:**
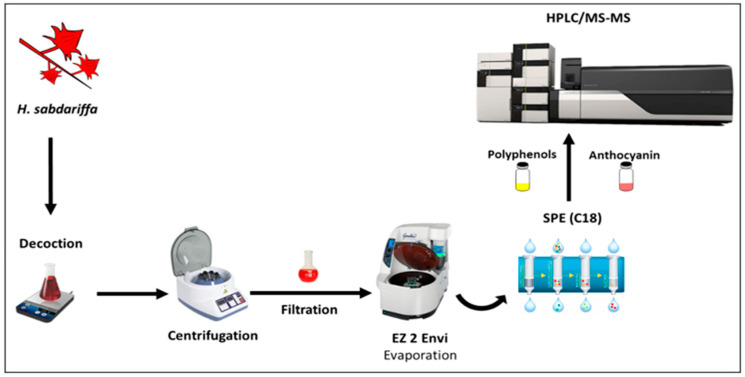
The extraction procedure of *H.s.* dried calyces and its analysis by liquid chromatography.

The extraction of the calyces of *H.s.* was prepared for in vitro simulated human digestion (Figure 1). Ground *H.s.* calyces (10 g (*w/w*)) were weighed (dry matter of 93% (RSD = 0.11%)) and placed into the Erlenmeyer flask with cold distilled water. After decocting for 10 min and filtering through a muslin cloth in 250 mL conical flasks, the filtrate was centrifuged at 2060× *g* for 10 min and subsequently filtered through a 0.45 μmAcrodisc nylon membrane (Merck Life Science, Merck KGaA, Darmstadt, Germany) [6]. The obtained aqueous extract was dried and stored for 48 h at +4 °C in the dark prior to in vitro simulated digestion and HPLC-PDA/MS analysis.

### 2.3. Simulated Human Digestion

The aim of this procedure was to digest the *H.s.* polyphenol extract in the upper GIT under a simulated model of the human stomach (gastric digestion) and small intestine (duodenal digestion) [12].

#### 2.3.1. Gastric Digestion

Dried extracts of *H.s.* (1 g) were placedinto 50 mL falcon plastic tubes and dissolved in a simulated gastric acid solution (10 mL) containing NaCl (58 mM), KCl (30 mM), CaCl_2_ (0.5 mM), NaH_2_PO_4_ (0.864 mM), and egg-phosphatidylcholine (0.127 mM). The pH of the gastric solution was adjusted to 2.5 by adding 1 M HCl and porcine gastric mucosa pepsin and a gastric lipase were then dissolved at a final concentration of 9000 U mL^−1^ and 60 U mL^−1^, respectively. Samples were incubated for 2 h at +37 °C under constant agitation (170 rpm) in an Innova 4000 Benchtop Incubator Shaker (New Brunswick Scientific, Edison, NJ, USA). Two aliquots were collected every 20 min during gastric digestion. Gastric reactions were terminated by raising the pH to 7.5 by 1 M NaOH. Subsequently, the gastric digestion output was divided into two aliquots (5 mL each) and the first was transferred to the duodenal digestion while the other aliquot was filtered through a 0.45 μm membrane filter and retained for analyses.

#### 2.3.2. Duodenal Digestion

The second aliquot of gastric output (5 mL containing about 0.5 g of extract) was transferred to another 50 mL falcon plastic tubes for 30 min under duodenal digestion conditions, with the addition of simulated bile solution (4.33 mL, containing 12.5 mM sodium taurocholate, 12.5 mM sodium glycodeoxycholate, 6.5 mM dried lecithin, and 4 mM cholesterol) and pancreatic enzyme solution (12.17 mL, consisting of NaCl (125.0 mM), CaCl_2_ (0.6 mM), MgCl_2_ (0.3 mM), ZnSO_4_·7H_2_O (4.1 μM), porcine pancreatic lipase (590 U mL^−1^), porcine colipase (3.2 U mL^−1^), porcine trypsin (11 U mL^−1^), bovine α-chymotrypsin (24 U mL^−1^), and porcine α-amylase (300 UmL^−1^). The mixture was incubated at 37 °C under shaking conditions (170 rpm) for 30 min. During duodenal digestion, an aliquot was harvested every 10 min. The different post duodenal aliquots were filtered separately through a 0.45 μm membrane filter and stored at −80 °C for analyses.

#### 2.3.3. Polyphenols Extraction after Simulated Digestion

**Figure 2:**
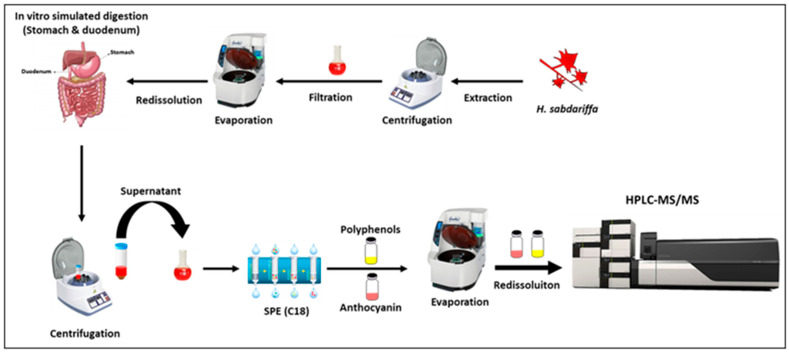
Extraction of the phenolic compounds of *H.s*. after in vitro human simulated digestion.

All the obtained aliquots from different samples during gastric and duodenal simulated digestion were collected and centrifuged to separate the residual material from polyphenols present in the supernatant. Supernatants of *H.s.* extract and aliquots were applied to the solid phase extraction (SPE; Sep-Pak Vac C18 Octadecyl cartridge (3 mL, 200 mg), VWR International Srl, Milan, Italy) after evaporation close to dry by Ez-2 and redissolved in 1 mL of acidified milli-Q water (3 times 0.1% Formic acid) to dispose undesirable products (e.g., proteins, enzymes, and carbohydrates) following the procedure already cited in the previous study [6] (Figure 2).

### 2.4. Polyphenolic Compounds Analysis by HPLC-PDA-MS/MS

#### 2.4.1. Instrumentation and Software

The HPLC-PDA-MS/MS analyses were carried out using a Shimadzu Prominence LC-20A (Shimadzu, Kyoto, Japan), consisting of a CBM-20A controller, two LC-20AD dual-plunger parallel-flow pumps, a DGU-20 A5 degasser, a SPDM20A photodiode array detector, a CTO-20AC column oven at 25 °C, a SIL-20A auto-sampler and an Ascentis Express C18 column (150 × 4.6 mm, 2.7 µm; Merck Life Science, Merck KGaA, Darmstadt, Germany). LCMS-8050 triple quadrupole mass spectrometer equipped with electrospray ionization (ESI) source was used in positive and negative ionization modes. Data acquisition was obtained using a Shimadzu LabSolutions software (Ver. 5.65, Shimadzu, Kyoto, Japan).

MS and MS/MS parameters were previously reported [6].

#### 2.4.2. Calibration Curves and Limits of Detection (LoD) and Quantification (LoQ)

The employed commercially available standards included the following: cyanidin-3-*O*-glucoside, weighed and dissolved in methanol with 0.1% HCl [13]; caffeic acid, quercetin, coumarin, and kaempferol, dissolved in methanol. All stock standard solutions (1000 mg L^−1^) were prepared at six concentrations. Triplicate injections were made for each level and a linear regression was generated. All cyanidin-3-*O*-sambubioside and delphinidin-3-*O*-sambubioside were recorded within the linear range of the standard curve of cyanidin-3-*O*-glucoside with R_2_ = 0.998. The anthocyanin compounds were semi-quantified at a wavelength of 520 nm and expressed in µg g^−1^ of dried extract.

Validation of the HPLC analytical method for cyanidin-3-*O*-glucoside as an external standard compound of anthocyanins was obtained with triplicate injections in the range of 1–100 mg mL^−1^ for cyanidin-3-*O*-glucoside. The LoD and LoQ values were obtained using a standard deviation of blank response and slope of 3 and 10, respectively.

#### 2.4.3. Analysis of *H.s.* Anthocyanins

The mobile phase consisted of water/formic acid (90:10 *v/v*, solvent A) and water-acetonitrile–formic acid (40:50:10 *v/v/v*) (solvent B) with the following gradient: 0 min, 12% B; 35 min, 30% B; 36 min, 100% B [14]. PDA acquisition was in the range of 200–550 nm; the *H.s.* anthocyanins in aqueous extracts were monitored at 520 nm (sampling frequency: 12.5 Hz; time constant: 0.08 s). The injection volume of the anthocyanins was 5 µL.

#### 2.4.4. Analysis of *H.s.* Polyphenols

The mobile phase containing water/formic acid (99.9/0.1 *v/v*, solvent A) and acetonitrile (solvent B) was used with the following gradient: 0 min, 0% B; 5 min, 5% B; 15 min, 10% B; 30 min, 20% B; 60 min, 50% B; and 70 min, 100% B. The flow rate of 1 mL min^−1^ was split by a T-piece to 0.2 mL min^−1^ after PDA and before MS detection. The injection volume was 5 µL. PDA acquisition was in the range of 200–400 nm (sampling frequency: 12.5 Hz; time constant: 0.08 s).

### 2.5. Antimicrobial Assays

The antimicrobial potential of the *H.s.* polyphenols extract was assessed against a range of human bacterial pathogens.

#### 2.5.1. Microbial Strains and Culture Conditions

A range of Gram-positive and Gram-negative bacterial strains obtained from the University of Messina’s in-house culture collection (Messina, Italy) was used: *Staphylococcus aureus* ATCC 6538; *Escherichia coli* ATCC 10536; *Salmonella typhimurium* ATCC 14028; *Bacillus subtilis* ATCC 6633; *Enterococcus hirae* ATCC 10541; *Listeria monocytogenes* ATCC 7644; 16 food-isolated strains of *Listeria monocytogenes* belonging to serotypes 1/2a (10 strains) and 1/2b (6 strains). All strains were grown in Mueller-Hinton Broth (MHB, Oxoid, CM0405) at 37 °C (18–20 h) for the susceptibility studies.

#### 2.5.2. Minimum Inhibitory Concentration (MIC) and Minimum Bactericidal Concentration (MBC)

The minimum inhibitory concentration (MIC) and the minimum bactericidal concentration (MBC) of *H.s.* extract were determined by the broth microdilution method according to CLSI [15]. The MIC was defined as the lowest concentration which completely inhibited bacterial growth after 20 h. The MBCs were determined by seeding 20 μL from all clear MIC wells onto Mueller-Hinton agar (MHA, Oxoid) plates. The MBC was defined as the lowest extract concentration that killed 99.9% of the final inoculum after 24 h incubation.

### 2.6. Statistical Analysis

Normality of the data and of the residuals was checked using the Shapiro–Wilk test and heteroscedasticity was checked using the white test after carrying out a nonparametric regression. The Friedman’s test (a nonparametric repeated measures comparisons) was performed owing to the absence of a normal distribution for comparing the effect of the phase and time of digestion on the release of polyphenols. When statistically significant differences were detected, a post hoc analysis was conducted using Dunnett’s and Nemenyi’s tests. Statistically significant effects were accepted at the 95% level. Data are presented as means ± SD. All statistical analyses were performed in Xlstat (version 2019.2.2).

## 3. Results and Discussion

### 3.1. Identification of Polyphenols in the Extract of H.s.

**Figure 3:**
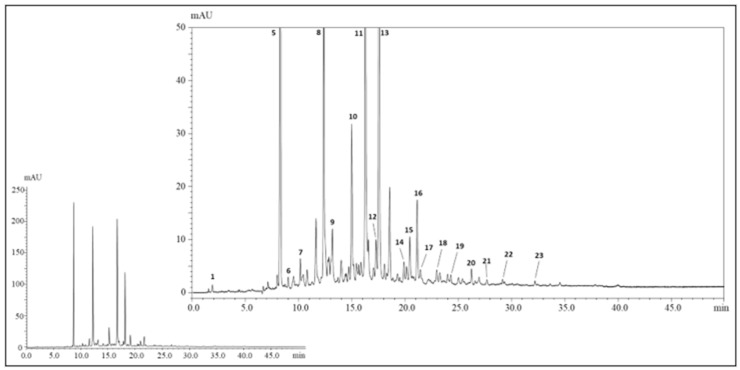
Profile of polyphenolic compounds in the aqueous extract of dried calyces of *H.s.* (λ = 280 nm). The inset illustrates the unzoomed chromatogram.

The polyphenolic profile of *H.s.* has been investigated in previous studies [16,17,18,19,20,21,22,23]; however, until now no characterization studies have been performed for such a species grown in Morocco. Figure 3 shows the polyphenolic profile of dried calyces of *H.s.*, achieved by HPLC-PDA-MS/MS. In total, up to twenty-three polyphenolic compounds were detected and among them twenty-one were positively identified according to retention times, MS, and literature data (Table 1). Peak no. 1 (t_R_ = 1.74 min, λ_max_ = 260) was identified as hibiscus acid, based on the UV-vis spectrum, deprotonated molecule [M-H]^−^ at *m/z* 189, and mass fragment at *m/z* 127 derived from the typical losses of water and carbon dioxide from the main ion. Such a compound was reported in all previous studies and it represents a lactone of the hydroxycitric acid. Among phenolic acids, hydroxycinnamic ones are the most represented with twelve compounds positively identified (peaks no. 6–8,10,11,13–16, and 18–20). Peaks no. 8, 11, 13, and 14 reported a characteristic λ_max_ = 325, a deprotonated molecule [M-H]^−^ at *m/z* 353, and mass fragments at *m/z* 191 and 179 and were thus identified as caffeoylquinic acids; on the other hand peaks 10,15, and 16 showed a λ_max_ = 310 nm, a deprotonated molecule [M-H]^−^ at *m/z* 337, and a mass fragment at *m/z* 191 and were identified as coumaroylquinic acids. Concerning flavonoids, peaks no. 17,21,22, and 23 showed a λ_max_ = 319, 345, 353, and 350 nm with deprotonated molecules [M-H]^−^ at *m/z* 449, 595, 609, 593, 595, and 609, and mass fragments at *m/z* 317, 301, and 285 (corresponding to the loss of sugar moieties); such compounds were positively identified as myricetin-arabinoside, quercetin-sambubioside, quercetin-rutinoside, and kaempferol-rutinoside. Finally, peaks no. 3 and 4, λ_max_ = 520 nm, deprotonated molecules [M-H]^−^ at *m/z* 597 and 579, and mass fragments at *m/z* 303 and 287 were positively characterized as delphinidin-sambubioside and cyanidin-sambubioside.

### 3.2. Release of Phenolic Compounds and Flavonoids from H.s. during In Vitro Digestion

A decrease in certain compounds was observed post in vitro gastric and gastric + duodenal digestion. In particular, peaks no. 8, 11, 13, and 14 identified as caffeoylquinic acids partially decreased starting after 20 min of gastric digestion. Likewise, peaks no. 10, 15, and 16 identified as coumaroylquinic acids only persisted little throughout the simulated human digestion as a result of the degradation occurring in the gastric and duodenal compartment, as previously reported [8]. On the other hand, as reported in Table 1, quinic acid was only present in the samples obtained during duodenal digestion, suggesting how this compound was derived from the degradation of the more complex caffeoylquinic and coumaroylquinic acids. As expected, hibiscus acid was present in the undigested extract and persisted, albeit in lower concentrations, throughout the simulated digestion. Interestingly, all peaks eluted on or after 23 min were completely solubilized after 20 min gastric incubation.

**Figure 4:**
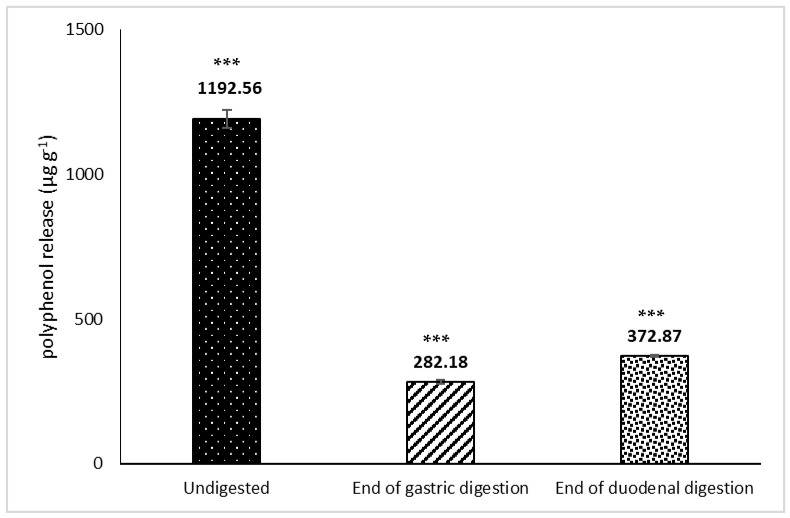
Evaluation of total polyphenols content in the extract at the end of gastric and duodenal digestion. Values are given as the amount of polyphenols in the undigested extract (baseline) and in the soluble extract released during in vitro gastric and gastric + duodenal digestion. Values represent averages (±SD) of triplicate measurements. RSD was always <10%. Statistically significant differences were observed (Friedman’s test followed by post hoc comparison with one-tailed Dunnett’s test) and are characterized by the * symbol. Statistically significant differences were observed between the end of gastric and duodenal digestions and undigested extract (*p* < 0.001) (***).

**Figure 5:**
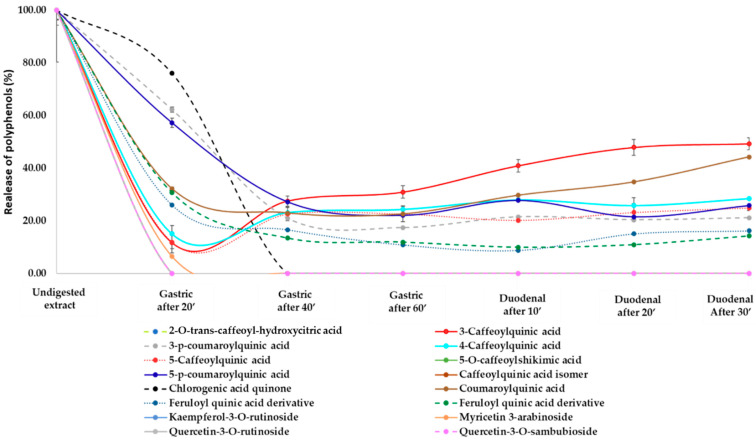
Dynamic evaluation of the release of polyphenolic compounds content during the in vitro simulated human digestion. Values represent averages (±SD) of triplicate measurements. RSD was always <10%.

The majority of the polyphenols from *H.s.* was released in the stomach (23%), with a slight increase during the gastric + duodenal digestion (31%, Figure 4). This can be explained by the rapid loss of polyphenols from the gastric compartment, after which the rate of release was reduced (Figure 5). We have previously shown similar trends of lipid, protein, and vitamin E release from almond seeds during digestion, with the cell walls playing an important role in regulating bioaccessibility [11]. More than 90% of the polyphenols from pistachio seeds were released in the gastric compartment using a dynamic gastric model of digestion, with virtual total release in the duodenal phase [10].

A major challenge understanding the role of individual health promoting components in natural extracts is the lack of data on their behavior in the GIT together with their bio-transformation by the gut microbiota enzymes [7]. The influence of digestion conditions, such as pH, temperature, bile salts, gastric, and pancreatic enzymes on the bioaccessibility of certain polyphenols has also been reported [24,25]. Due to the ability of polyphenols to bind proteins, possible protein denaturation (e.g., α-amylase, trypsin, lysozyme) may result in lower digestibility in the upper GIT and protection from oxidative reactions in the large bowel [26,27]. Therefore, the health benefits associated with polyphenols intake depend on a large number of factors related to their release in the upper GIT, a possible effect played by the food matrix on their release as well as degree of bacterial fermentation in the large bowel [28].

### 3.3. Release of Anthocyanins from H.s. during In Vitro Digestion

**Figure 6:**
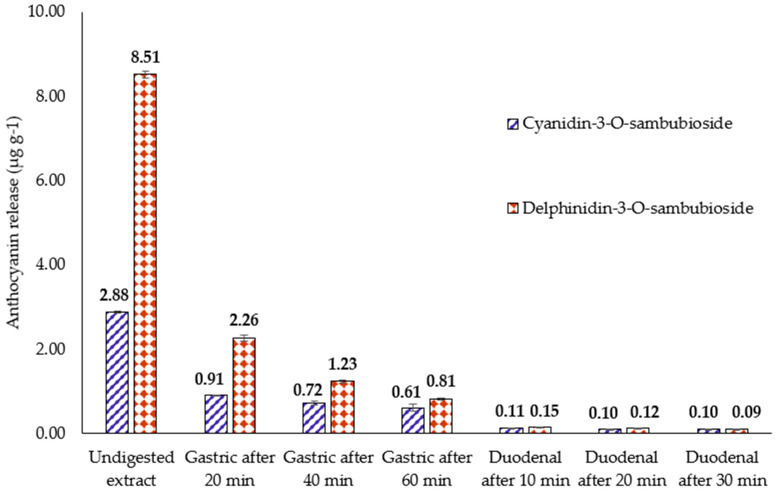
Quantification of anthocyanins in the raw extract and during in vitro gastric and duodenal digestion.

Anthocyanins, mainly delphinidin-sambubioside and cyanidin-sambubioside, represent, with organic acids, polysaccharides and flavonoids, the principal components of *H.s.* which are relevant for food and pharmaceutical industries. Figure 6 reports the quantification of delphinidin-3-sambubioside and cyanidin-3-sambubioside in the *H.s.* undigested extract and during simulated human digestion. As for the flavonoids release, the majority of anthocyanins were bioaccessible in the gastric compartment, with a slight further increase in the duodenal phase. A total of 49% (26% after 20 min gastric phase, 14% after 40 min gastric phase, and 9% after 60 min gastric phase) and 77% (31% after 20 min gastric phase, 25% after 40 min gastric phase, and 21% after 60 min gastric phase) of delphinidin-3-sambubioside and cyanidin-3-sambubioside werereleased in the gastric compartment, respectively. A further 3% and 10% of delphinidin-3-sambubioside and cyanidin-3-sambubioside, respectively, weresolubilized in the small intestine. A recent investigation reported on the release of anthocyanins from a hibiscus extract encapsulated by ionic gelation: the results demonstrated that the application of microparticles in jelly candy proved to be feasible, with retention of up to 73% of bioactive compounds [29].

In the present study, the anthocyanin release trend was similar to the one observed for polyphenol release in terms of gastric and duodenal distribution, but at higher release values. On the contrary, de Moura et al. [29] have reported lower release values for anthocyanins compared with polyphenols. The observed differences may be related to solubility and the enzymes used. Eker et al. [30] reviewed the factors affecting anthocyanin bioavailability and analyzed the possible implications for the inter-individual variability: Although anthocyanins are dietary bioactive compounds with a range of beneficial effects against cardiovascular, neurological, and eye conditions, factors including food matrix and food processing, enzymes involved in their metabolism and transport, and anthocyanin metabolizing gut microbiota may be responsible for the high intra-variability and inter-variability in bioaccessibility studies.

### 3.4. Antimicrobial Studies

The Minimum Inhibiting Concentration (MIC) and Minimum Bactericidal Concentration (MBC) values of the *H.s.* extract against the strains tested are shown in Table 2. Amongst the Gram-positive strains, the extract was active against *S. aureus* ATCC 6538 (MIC values of 2.5 mg mL^−1^) and a food isolate of *Listeria monocytogenes* (MIC values of 2.5 mg mL^−1^), whereas no activity was found against the Gram-negative bacteria at the concentrations tested. The effect was bacteriostatic rather than bactericidal with the exception of a food isolate of *Listeria monocytogenes*, where the MBC values werethe same as the MIC (2.5 mg mL^−1^).

A couple of studies have previously reported the antimicrobial potential of *H.s.* [31,32]. Baena-Santillán et al. [31] have recently determined and compared the antimicrobial effect of *H.s.* calyx extracts, six types of commercial mouthwashes, and chlorhexidine on a number of Gram-positive strains including *Streptococcus mutans*, *Streptococcus sanguinis*, *Capnocytophaga gingivalis*, and *Staphylococcus aureus*: their results showed that the extract was able to alter the permeability of the bacterial membranes. The *H.s.* extract was also able to inhibit the adhesion, biofilm initiation, and formation of the yeast *Candida albicans* [32]. We have previously demonstrated the effect of a plant extract against the Gram-positive strains *Staphylococcus aureus* and *Listeria monocytogenes* [33,34]. The present study confirmed that Gram-positive strains were more susceptible to plant extracts than Gram-negative bacteria. Amongst the Gram-positive human pathogens, *S. aureus* is responsible for a number of infections, including skin, respiratory, and bone joint infection as well as endocarditis, bacteremia, and toxic shock syndrome [35]. Due to the increased spread of antibiotic resistance globally, more effort is focused on novel antimicrobial agents against *S. aureus* and MRSA. The data obtained in this study are promising for the identification of novel strategies to combat antibiotic resistance.

## 4. Conclusions

In this work, it was demonstrated that the polyphenolic compounds in *Hibiscus sabdariffa* become rapidly accessible in the stomach, maximizing the possibility of absorption in the upper small intestine and thus contributing to the beneficial relation between hibiscus consumption and health-related outcomes. Further human clinical studies are needed to validate these in vitro findings on the release of bioactives from hibiscus, as well as on the metabolism of the undigested polyphenols by the gut microbiota.

## Figures and Tables

**Table 1:** Identification of the available polyphenolic compounds in the extract of *H.s.* during simulated human digestion. x indicates the presence of the compound in the *H.s*. extract.—indicates absence of the compound in the extract.

Peak	t_R_ (min)	λ_max_ (nm)	[M-H]^−^; MS/MS	Tentative Identification	Undigested Extract	Gastric Digestion			Duodenal Digestion			Refs.
						20 min	40 min	60 min	10 min	20 min	30 min	
1	1.74	260	189,127	Hibiscus acid	×	×	×	×	×	×	×	[16,17,18,19,20,21,22,23]
2	3.53	280	387	Quinic acid	-	-	-	-	×	×	×	
3	4.03	520	597,303 *	Delphinidin-sambubioside	×	×	×	×	×	×	×	[16,17,18,19,20,21,23]
4	6.35	520	579,287 *	Cyanidin-sambubioside	×	×	×	×	×	×	×	[16,17,18,19,20,21,23]
5	8.11	286	235	Hibiscus acid hydroxyethylester	×	×	-	-	-	-	-	[17]
6	8.89	314	315	Chlorogenic acid quinone	×	×	-	-	-	-	-	[17]
7	10.15	317	369,191	Caffeoyl-hydroxycitric acid	×	-	-	-	-	-	-	[17,19]
8	12.34	325	353,191,179	Caffeoylquinic acid	×	×	×	×	×	×	×	[21,22,23]
9	13.13	287	297	Unknown	×	-	-	-	-	-	-	-
10	15.06	310	337, 191	Coumaroylquinic acid	×	×	×	×	×	×	×	[17,19]
11	16.28	325	353,191,179	Caffeoylquinic acid isomer	×	×	×	×	×	×	×	[20,21,22,23]
12	17.23	326	367	Unknown	×	×	×	×	×	×	×	-
13	17.61	325	353,191,179	Caffeoylquinic acid isomer	×	×	×	×	×	×	×	[20,21,22,23]
14	19.74	325	353,191,179	Caffeoylquinic acid isomer	×	-	-	-	-	-	-	[22]
15	20.58	309	337,191	Coumaroylquinic acid isomer	×	×	×	×	×	×	×	[19,21]
16	21.16	309	337,191	Coumaroylquinic acid isomer	×	×	×	×	×	×	×	[19,21]
17	21.38	352	449,317	Myricetin-arabinoside	×	×	-	-	-	-	-	[16,18,19]
18	22.97	329	367,193	Feruloylquinic acid derivative	×	×	×	×	×	×	×	[20]
19	24.33	326	335,179	Caffeoylshikimic acid	×	-	-	-	-	-	-	[16,17,18,19,21]
20	26.29	329	367,193	Feruloylquinic acid derivative	×	×	×	×	×	×	×	[20]
21	27.88	345	595,301	Quercetin-sambubioside	×	-	-	-	-	-	-	[16,17,18,19,21]
22	29.18	353	609,301	Quercetin-rutinoside	×	-	-	-	-	-	-	[16,17,18,19,21,22,23]
23	32.27	350	593,285	Kaempferol-rutinoside	×	-	-	-	-	-	-	[16,17,18,19,21,23]

* Acquired in [M+H]^+^.

**Table 2:** MICs and MBCs of *H.s.* extract (expressed as mg mL^−1^) against Gram-positive bacteria and Gram-negative bacteria.

Strain	MIC	MBC
*Staphylococcus aureus* ATCC 6538	2.5	>2.5
*Escherichia coli* ATCC 10536	>2.5	>2.5
*Salmonella typhimurim* ATCC 14028	>2.5	>2.5
*Bacillus subtilis* ATCC 6633	>2.5	>2.5
*Enterococcus hirae* ATCC 10541	>2.5	>2.5
*Listeria monocytogenes* ATCC 7644	>2.5	>2.5
*Listeria monocytogenes* (food isolate)	>2.5	>2.5
*Listeria monocytogenes* (food isolate)	>2.5	>2.5
*Listeria monocytogenes* (food isolate)	2.5	2.5
*Listeria monocytogenes* (food isolate)	>2.5	>2.5
*Listeria monocytogenes* (food isolate)	>2.5	>2.5
*Listeria monocytogenes* (food isolate)	>2.5	>2.5
*Listeria monocytogenes* (food isolate)	>2.5	>2.5
*Listeria monocytogenes* (food isolate)	>2.5	>2.5
*Listeria monocytogenes* (food isolate)	>2.5	>2.5
*Listeria monocytogenes* (food isolate)	>2.5	>2.5
*Listeria monocytogenes* (food isolate)	>2.5	>2.5
*Listeria monocytogenes* (food isolate)	>2.5	>2.5
*Listeria monocytogenes* (food isolate)	>2.5	>2.5
*Listeria monocytogenes* (food isolate)	>2.5	>2.5
*Listeria monocytogenes* (food isolate)	>2.5	>2.5
*Listeria monocytogenes* (food isolate)	>2.5	>2.5
*Listeria monocytogenes* (food isolate)	>2.5	>2.5
*Listeria monocytogenes* (food isolate)	>2.5	>2.5

MICs, minimal inhibitory concentrations; MBCs, minimal bactericidal concentrations.

## Data Availability

Available under authors request.
